# TIM‐3 expression induces resistance to PD‐1 inhibitor in G‐CSF‐producing lung spindle cell carcinoma: A case report

**DOI:** 10.1111/1759-7714.15149

**Published:** 2023-11-05

**Authors:** Fumiko Hayashi, Kazumasa Akagi, Hirokazu Taniguchi, Toyoshi Matsutake, Hiromi Kawahara, Ichiro Sekine, Hiroshi Gyotoku, Shinnosuke Takemoto, Hiroshi Soda, Kazuto Ashizawa, Hiroshi Mukae

**Affiliations:** ^1^ Department of Respiratory Medicine Nagasaki University Hospital Nagasaki Japan; ^2^ Department of Respiratory Medicine Nagasaki University Graduate School of Biomedical Sciences Nagasaki Japan; ^3^ Clinical Oncology Center Nagasaki University Hospital Nagasaki Japan; ^4^ Department of Respiratory Medicine Kouseikai Hospital Nagasaki Japan; ^5^ Department of Internal Medicine Kouseikai Hospital Nagasaki Japan; ^6^ Department of Pathology Kouseikai Hospital Nagasaki Japan; ^7^ Department of Respiratory Medicine Sasebo City General Hospital Sasebo Japan; ^8^ Department of Clinical Oncology Nagasaki University Graduate School of Biomedical Sciences Nagasaki Japan

**Keywords:** acquired resistance, immune checkpoint blockade, M2 macrophage, spindle cell carcinoma, TIM‐3

## Abstract

Lung spindle cell carcinoma is an aggressive subtype of pleomorphic lung cancer resistant to cytotoxic chemotherapy. Programmed cell death‐1 (PD‐1) inhibitors have been reported to have clinical effects in patients with spindle cell carcinoma; however, the resistance mechanism to PD‐1 inhibitors is yet to be fully elucidated. Herein, we report the case of an 88‐year‐old man with G‐CSF‐producing spindle cell carcinoma who acquired resistance to PD‐1/PD‐ligand 1 (L1) inhibitor in an early setting after a remarkable response. A histopathological review of the resistant specimen revealed a low count of CD8^+^ T cells and a predominant presence of M2 and TIM‐3^+^ macrophages, indicating the presence of an immunosuppressive microenvironment. Our findings suggest a novel resistance mechanism to PD‐1/PD‐L1 inhibitors in G‐CSF‐producing spindle cell carcinoma.

## Graphical abstract

In G‐CSF producing lung spindle cell carcinoma, oversecreted G‐CSF encourages M2 macrophages and Tim‐3^+^ macrophages to proliferate, which may represent a new resistance mechanism to PD‐1/PD‐L1 inhibitors.

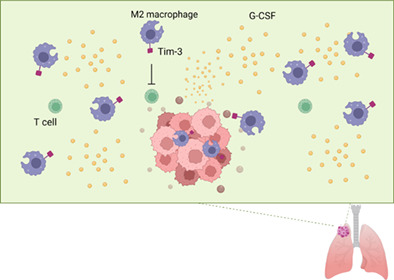

## INTRODUCTION

Lung spindle cell carcinoma is a rare subtype of pleomorphic carcinoma and a subset of sarcomatoid carcinoma (SC).^1^ The prognosis of pleomorphic carcinoma is worse than that of other non‐small cell lung cancers (NSCLC). Therefore, a novel treatment strategy is urgently needed.

Immune checkpoint blockades (ICBs) are key drugs used to treat NSCLC.^2^, ^3^ A previous study reported that ICBs showed a clinical effect in pulmonary SC with a 40.5% overall response rate (ORR) and a 12.7‐month overall survival (OS).^4^ In addition, pleomorphic carcinomas have a high incidence of PD‐L1 expression (60%–90%).^5^ Other studies have shown that chemotherapy for advanced SC resulted in 0%–16.5% of ORR and 5–7.7 months of OS.^6^, ^7^ These results indicate that ICB can significantly improve the prognosis of SC compared with chemotherapy. However, most patients who receive ICB with a clinical response experience relapse because of acquired resistance.

Herein, we report a case of G‐CSF‐producing spindle cell carcinoma in which ICB was once effective but acquired early resistance. Immune cell profiles were comprehensively evaluated using immunohistochemical (IHC) examination of autopsy samples to explore resistance mechanisms to ICB.

## CASE REPORT

**FIGURE 1:**
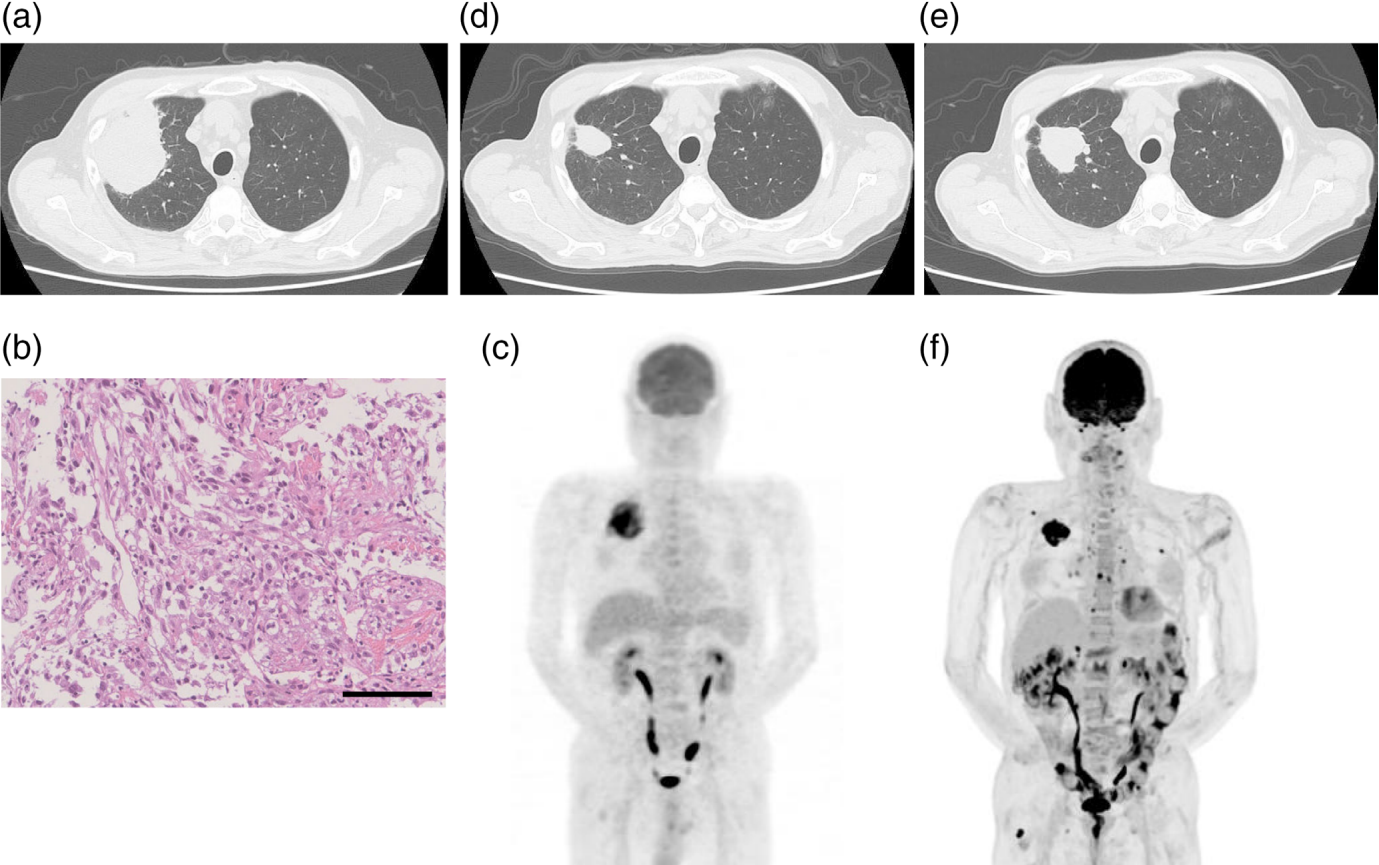
Change of chest computed tomography (CT) and positron emission tomography (PET)‐CT images and pathological findings in the patient with spindle cell carcinoma in pretreatment setting. (a) In pretreatment, the chest CT showed a large mass in the right upper lobe. (b) Pathological diagnosis of the lung tumor was spindle cell carcinoma (hematoxylin–eosin [H&E] staining). The scale bar indicates 100 μm. Original magnification of 10×. (c) PET‐CT showed remarkably high FDG uptake of the mass and diffuse uptake throughout the bone marrow. (d) Chest CT after seven cycles of pembrolizumab showed a decrease in the size of the tumor. (e) The tumor reincreased in size and (f) multiple metastases in bones and bilateral adrenal glands were clearly demonstrated in PET‐CT images after 11 cycles of the treatment, 8 months after the initiation of the treatment.

An 88‐year‐old man, an ex‐smoker, visited our department with back pain. Chest computed tomography (CT) showed a large mass measuring 80‐mm in diameter in the right upper lobe associated with pleural effusion (Figure 1a). He was eventually diagnosed with lung spindle cell carcinoma (cT4N0M1a, UICC version 8) without any driver mutations (Figure 1b). The PD‐L1 (22C3) tumor proportion score of the tumor was 100%. In ^18^F‐fluorodeoxyglucose (^18^F‐FDG) positron emission tomography (PET)‐CT, ^18^F‐FDG uptake was observed in the tumor and diffused throughout the bone marrow (Figure 1c). Leukocytosis (white blood cell count [WBC] 30.8 × 10^9^/L) was also observed, suggesting that the tumor produced G‐CSF. However, the serum G‐CSF level was within the normal upper limit of 38 pg/mL.

He received pembrolizumab (200 mg/body, triweekly) as first‐line treatment. The tumor had decreased in size and leukocytosis had improved after seven cycles of pembrolizumab treatment (Figure 1d). However, they relapsed after 11 cycles, 8 months after the initiation of treatment (Figure 1e, f). WBC and serum G‐CSF levels were elevated (16.6 × 10^9^/L and 93.5 pg/mL, respectively) again. Another ICB, atezolizumab, was added at the patient's request; however, 4 days later, black stools appeared with no abnormalities on gastroendoscopy or colonoscopy. He was diagnosed with autoimmune small intestine inflammation caused by ICB and underwent high‐dose methylprednisolone therapy and systemic management to treat autoimmune small intestine inflammation as an immune‐related adverse event (irAE), which could progress rapidly and be fatal. However, 50 days after the initiation of atezolizumab, the patient died due to multiple organ failure.

**FIGURE 2:**
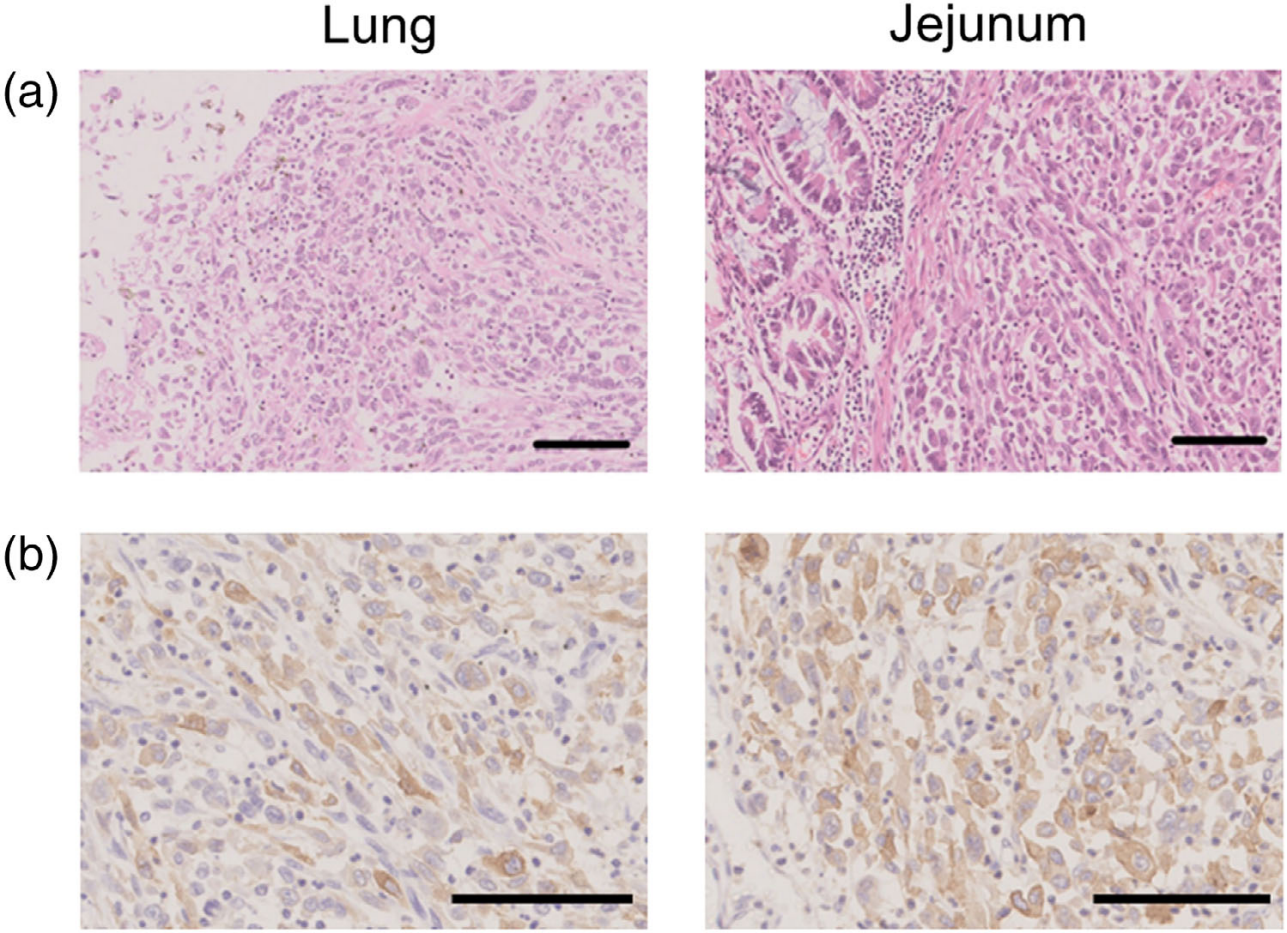
Pathological findings of lung (left) and small intestine (right) specimens by autopsy. (a) Hematoxylin‐eosin (HE) staining. (b) PD‐L1 (22C3) was positive in both samples. The scale bar indicates 100 μm. Original magnification of (a); 10×, (b); 20×.

**FIGURE 3:**
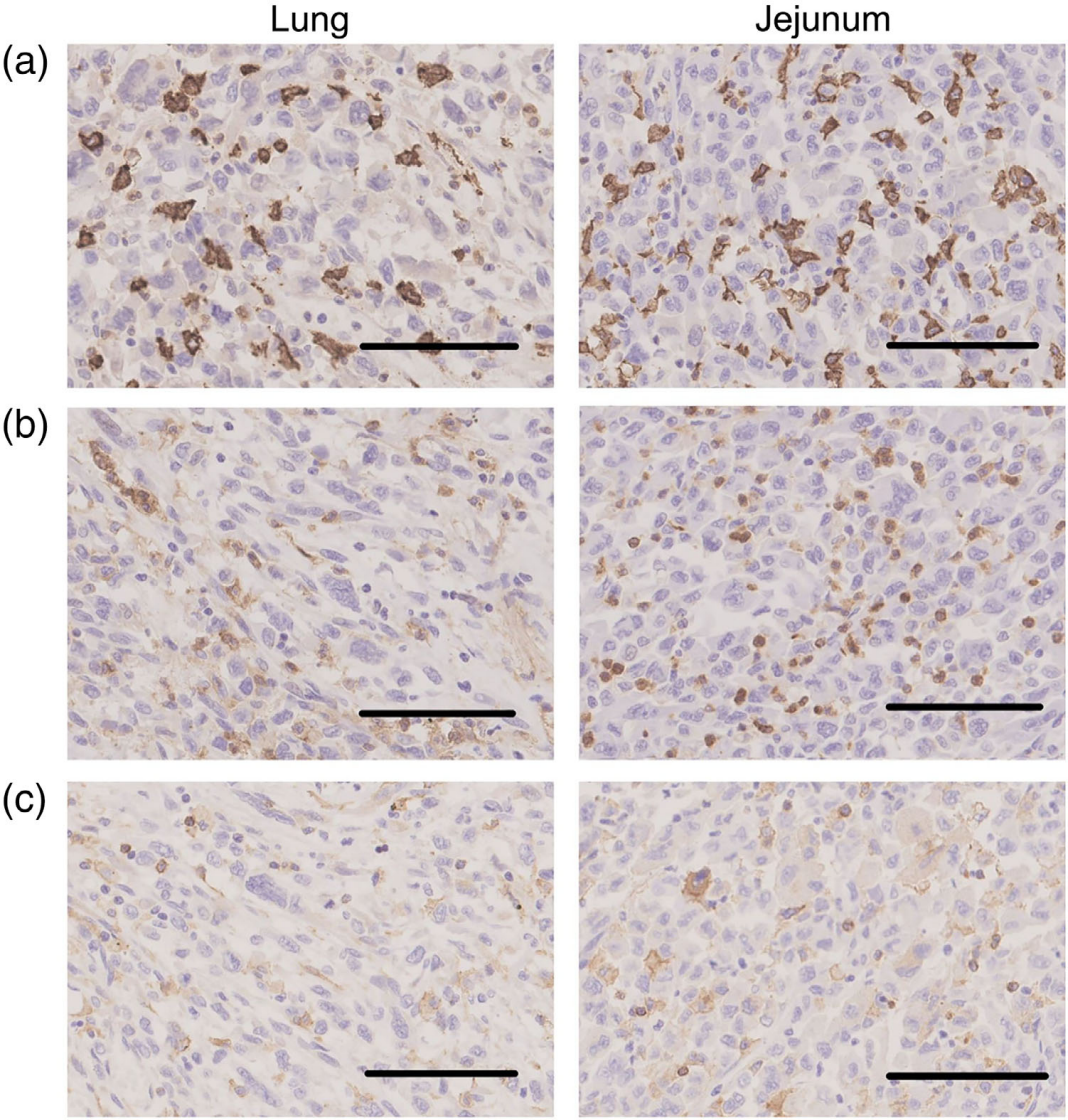
Pathological findings of lung (left) and small intestine (right) specimens by autopsy. (a) CD163^+^ cells, (b) CD11b^+^ cells, (c) TIM‐3^+^ cells in the tumor in both samples. The scale bar indicates 100 μm. Original magnification of 20×.

**FIGURE 4:**
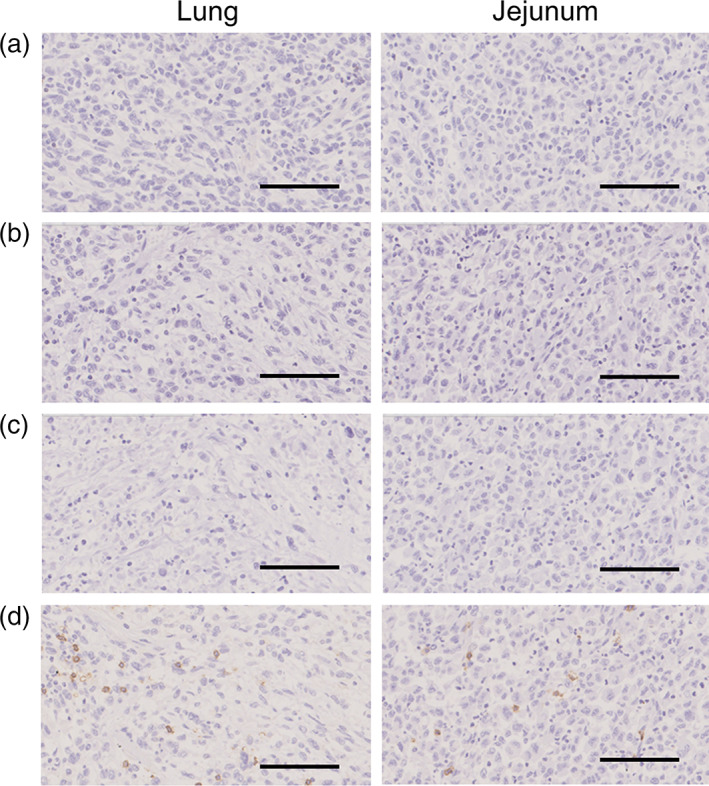
Pathological findings of lung (left) and small intestine (right) specimens by autopsy. (a) LAG3, (b) TIGIT, (c) FOXP3, and (d) CD8^+^ cells in the tumor in both samples. The scale bar indicates 100 μm. Original magnification of 20×.

An autopsy was performed with the consent of his family members, and multiple metastases and intussusceptions in the jejunum were confirmed instead of autoimmune inflammation. A histopathological review of the tumors in the lung and jejunum revealed that PD‐L1 was highly expressed in both samples (Figure 2a, b). CD163^+^ and CD11b^+^ macrophages infiltrated the tumors (Figure 3a, b). These cells were positive for TIM‐3 (Figure 3c) and negative for other immune checkpoint molecules, including LAG3, TIGIT, and FOXP3 (Figure 4a–c). Few CD8^+^ lymphocytes were observed in the tumor (Figure 4d).

## DISCUSSION

In this study, we observed a case with lung spindle cell carcinoma acquiring early resistance to ICB and highlight M2 and TIM‐3 positive macrophages as the resistance mechanism. ICB is an established first‐line treatment for patients with NSCLC harboring high PD‐L1 expression^2^, ^3^; however, acquired resistance after the initial response limits its clinical benefits. Several mechanisms of primary or acquired resistance to ICB have been reported, including the secretion of PD‐L1 variants,^8^ biallelic PTEN loss,^9^ allele‐specific HLA loss,^10^ and expression of other inhibitory immune checkpoint molecules, such as TIM‐3, TIGIT, and LAG‐3.^11^, ^12^

TIM‐3 is a type I transmembrane protein initially identified as a specific marker that negatively regulates helper and cytotoxic T cells by binding to its main ligand, galectin‐9.^13^ Its expression has subsequently been observed in other immune cells, including Tregs, macrophages, and dendritic cells.^14^ TIM‐3 negatively regulates the immune functions of macrophages and dendritic cells. TIM‐3 can also indirectly suppress immune responses by facilitating the generation of MDSCs via a TIM‐3/galectin‐9‐dependent mechanism.^15^ In the present case, a limited number of CD8^+^ cytotoxic T cells were observed, and TIM‐3^+^ macrophages were detected in the acquired resistant tumor, suggesting that TIM‐3‐positive cells might mediate the inactivation of antitumor immunity.

G‐CSF is a well‐known cytokine commonly used as a pharmaceutical agent to treat neutropenia, a side effect of chemotherapy. However, several cancers, including lung cancer, produce G‐CSF, and G‐CSF‐producing cancers exhibit poor prognoses and elevated mortality rates.^16^ A previous study reported that breast cancers exhibiting higher G‐CSF expression were associated with higher numbers of CD163^+^ M2 macrophages, which could suppress the antitumor immune response and result in a poor OS rate.^17^ In the present case, G‐CSF production may have induced early resistance to ICB by infiltrating CD163^+^ M2 macrophages.

This study had several limitations. The pretreatment specimen was very small; therefore, IHC evaluation could not be performed adequately with the specimen. In addition, IHC analysis of immune cells was not performed using multicolor immunostaining, which can simultaneously evaluate multiple immune markers. Also, although there were no abnormalities on the gastroendoscopy and colonoscopy, we retrospectively identified a small mass in the small intestine in the CT scan at the time the black stools appeared, which might have been indicative of a small intestine metastasis or the intussusception. Distinguishing the cause of intestinal bleeding during ICB treatment is challenging; however, our experience indicates that it is necessary to consider the possibility of small bowel metastasis or intussusception in addition to autoimmune small intestine inflammation as an irAE.

In conclusion, tumor‐infiltrating M2 macrophages expressing TIM‐3 may mediate the early acquisition of resistance to ICB in G‐CSF‐producing lung spindle cell carcinoma. Further studies with larger cohorts are needed to elucidate the mechanism underlying this population's acquired resistance to ICB.

## AUTHOR CONTRIBUTIONS

All authors had full access to the data in the study and take responsibility for the integrity of the data and the accuracy of the data analysis. Conceptualization: K.A., H.T., A.M., H.S. Resources: F.H., H.T., K.A., M.A., H.K., I.S., H.S. Supervision: H.T., H.S., K.A., H.M. Writing–original draft preparation: F.H., K.A., H.T. Writing–review and editing: H.T., H.G., S.T., H.S., H.M.

## CONFLICT OF INTEREST STATEMENT

The authors report no conflicts of interest related to this work.
